# Autologous Adipose-Derived Stem Cells Reduce Burn-Induced Neuropathic Pain in a Rat Model

**DOI:** 10.3390/ijms19010034

**Published:** 2017-12-22

**Authors:** Cen-Hung Lin, Sheng-Hua Wu, Su-Shin Lee, Yun-Nan Lin, Yur-Ren Kuo, Chee-Yin Chai, Shu-Hung Huang

**Affiliations:** 1 Division of Plastic Surgery, Department of Surgery, Kaohsiung Medical University Hospital, Kaohsiung Medical University, Kaohsiung 80708, Taiwan; gigilin119@msn.com (C.-H.L.); sushin@kmu.edu.tw (S.-S.L.); yunnan1123@gmail.com (Y.-N.L.); kuoyrren@gmail.com (Y.-R.K.); 2 Department of Anesthesia, Kaohsiung Medical University Hospital, Kaohsiung Medical University, Kaohsiung 80708, Taiwan; elsawu2@gmail.com; 3 Department of Surgery, School of Medicine, College of Medicine, Kaohsiung Medical University, Kaohsiung 80708, Taiwan; 4 Center for Stem Cell Research, Kaohsiung Medical University, Kaohsiung 80708, Taiwan; 5 Orthopaedic Research Center, Kaohsiung Medical University, Kaohsiung 80708, Taiwan; 6 Graduate Institute of Medicine, College of Medicine, Kaohsiung Medical University, Kaohsiung 80708, Taiwan; cychai@kmu.edu.tw; 7 Department of Pathology, Kaohsiung Medical University Hospital, Kaohsiung 80756, Taiwan

**Keywords:** adipose-derived stem cells, stem cell, burn scar, scar pain, neuropathic pain, anti-neuroinflammation, autophagy

## Abstract

Background: Burn scar pain is considered as neuropathic pain. The anti-inflammation and anti-neuroinflammation effects of adipose-derived stem cells (ASCs) were observed in several studies. We designed a study using a murine model involving the transplantation of autologous ASCs in rats subjected to burn injuries. The aim was to detect the anti-neuroinflammation effect of ASC transplantation and clarify the relationships between ASCs, scar pain, apoptosis and autophagy. Methods: We randomized 24 rats into 4 groups as followings: Group A and B, received saline injections and autologous transplantation of ASCs 4 weeks after sham burn, respectively; Group C and D, received saline injections and autologous transplantation 4 weeks after burn injuries. A designed behavior test was applied for pain evaluation. Skin tissues and dorsal horn of lumbar spinal cords were removed for biochemical analysis. Results: ASC transplantation significantly restored the mechanical threshold reduced by burn injury. It also attenuated local inflammation and central neuroinflammation and ameliorated apoptosis and autophagy in the spinal cord after the burn injury. Conclusion: In a rat model, autologous ASC subcutaneous transplantation in post-burn scars elicited anti-neuroinflammation effects locally and in the spinal cord that might be related to the relief of post-burn neuropathic pain and attenuated cell apoptosis. Thus, ASC transplantation post-burn scars shows the potential promising clinical benefits.

## 1. Introduction

Burn scar pain can be considered neuropathic as it can result in concurrent neuroinflammation and subsequent central sensitization [1]. The pain can be intractable; as yet, there is no definitive treatment [2].

Autologous fat grafting can improve the size and texture of burn scars, enhance angiogenesis, reduce inflammation and alleviate associated pain [3]. Notably, pain alleviation has been demonstrated in both murine and human studies [4,5]. Burn scar pain is experienced after 30–68% of burn injuries and can have a significant negative effect on the quality of life [1,6]. Autologous fat grafting has therefore garnered interest. In addition, clinical reports have described its ability to relieve some types of neuropathic pain [7,8].

In a previous study of fat grafting in rats, we demonstrated its simultaneous anti-inflammatory and pain relief effects on full-thickness thermal burn scars [9]. However, the mechanisms of these effects remain to be clarified. Several studies have observed anti-inflammation effects after the transplantation of adipose-derived stem cells (ASCs) in vitro and in vivo, with increased interleukin (IL)-10 and suppression of the NFκB signaling pathway [10,11,12,13,14,15]. Two of these studies also demonstrated anti-neuroinflammation effects [13,14]. However, no study has investigated ASC transplantation for post-burn neuropathic pain.

We previously observed the alleviation by fat grafting of spinal neuronal apoptosis in full-thickness thermal burn scars [9]. Autophagy has also gained much attention recently. By recycling intracellular debris, it can generate metabolic precursors that can promote survival in mammalian cells subjected to adverse conditions, for example through pathways promoted by Beclin 1 and microtubule-associated protein-1 light chain-3B (LC3B) [16,17]. However, it remains to be clarified how ASCs interrelate with apoptosis and autophagy. Based on our previous murine model [9,18], therefore, we designed a study involving the transplantation of autologous ASCs and the biochemical analysis of inflammation, apoptosis and autophagy in the skin and spinal cords of rats subjected to burn injuries. The aim was to detect the anti-neuroinflammation effect of ASC transplantation in post-burn scars, clarifying the relationships between ASCs, apoptosis and autophagy.

## 2. Results

### 2.1. Increased Mechanical Threshold after ASC Transplantation in Post-Burn Scar

**Figure 1:**
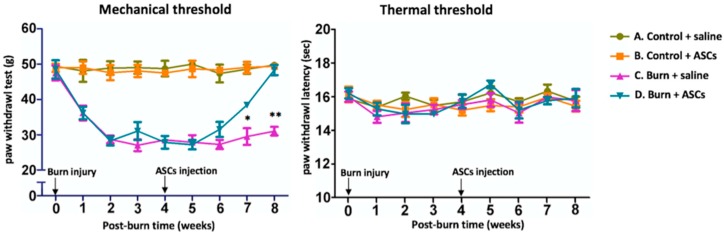
Increased mechanical threshold after ASC transplantations. Markedly decreased mechanical thresholds were observed after the burn injury at (i.e., in Groups C and D). The mechanical threshold had significantly improved in Group D at 3 weeks after ASC transplantation (4 weeks post-burn injury). The thermal thresholds of the four groups did not differ significantly within the 8-week study period. Each group contained six animals. Data are plotted as means ± standard errors of the means. (** *p* < 0.01 and * *p* < 0.05).

The burn groups, C (Burn + Saline) and D (Burn + ASCs), initially exhibited similar marked post-burn decreases in mechanical thresholds relative to the sham burn groups (A and B). However, 3 weeks after ASC transplantation, Group D exhibited a significant increase in the mechanical threshold relative to Group C (*p* < 0.05) that was also sustained at week 8 (*p* < 0.01) (Figure 1). This indicated that ASC transplantation significantly restored the mechanical threshold reduced by burn injury. There were no significant differences between the groups in thermal thresholds during the 8-week evaluation period.

Attenuated local inflammation and central neuroinflammation after ASC transplantation in post-burn scar.

**Figure 2:**
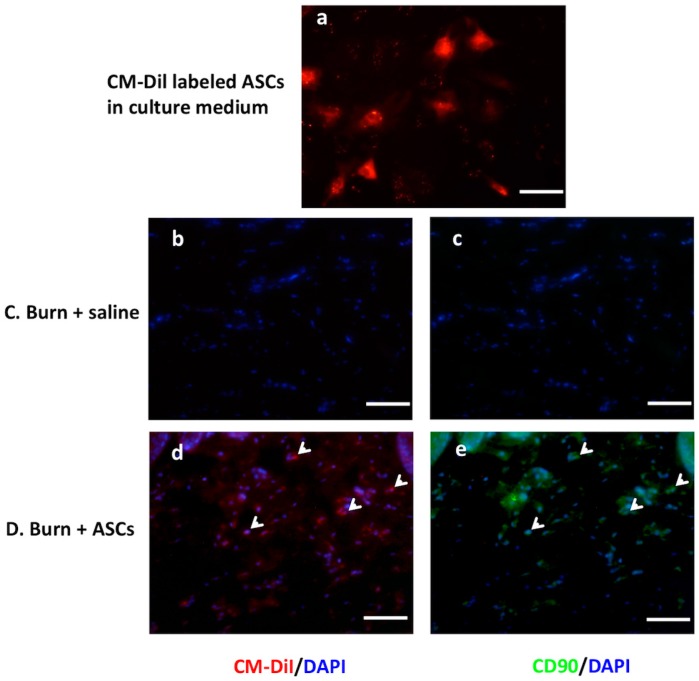
Engraftment of cultured ASCs into the post-burn dermis. (**a**). CellTracker CM-Dil-labeled cells (red spots) were detected among the cultured rat ASCs in culture medium at passage 4 before harvesting for treatment; (**b**,**c**) no double staining of CM-Dil/DAPI (blue) and CD90/DAPI detected in the same field within dermis 4 weeks after subcutaneous saline injection in Group C; (**d**,**e**) double staining of CM-Dil (red)/DAPI (blue) and CD90 (green)/DAPI in the same field (arrowheads) indicate the viable ASCs within the dermis 4 weeks after cultured ASC transplantation in Group D (fluorescence microscopic images; magnification, ×200).

Four weeks after transplantation (week 8), fluorescent microscopic analysis of the double staining of CM-Dil/DAPI and CD90/DAPI in the same field showed ASC engraftment to the dermal layer of the burned hind-paw in Group D (Figure 2d,e).

**Figure 3:**
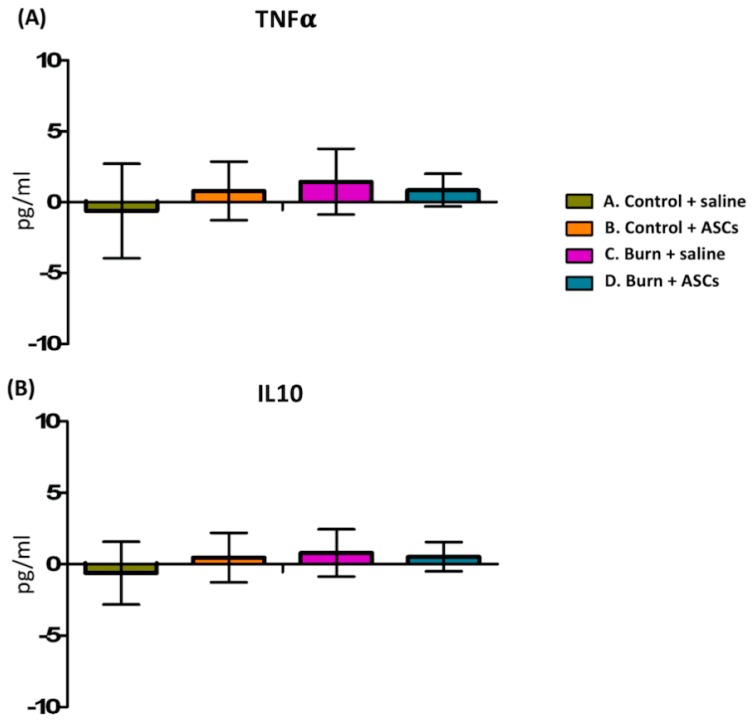
Insignificant difference of TNF-α and IL-10 levels at week 8 in serum among four groups.

In one of our previous studies using the same model, there was no significant change in serum TNF-α and INF-γ levels (inflammatory markers) at day 1 and 4 and week 8 after burn injury [19]. These absent systemic inflammatory effects might be related the small area of burn. In this study, we also found insignificant difference of TNF-α and IL-10 levels (inflammatory markers) at week 8 in serum among four groups (Figure 3).

**Figure 4:**
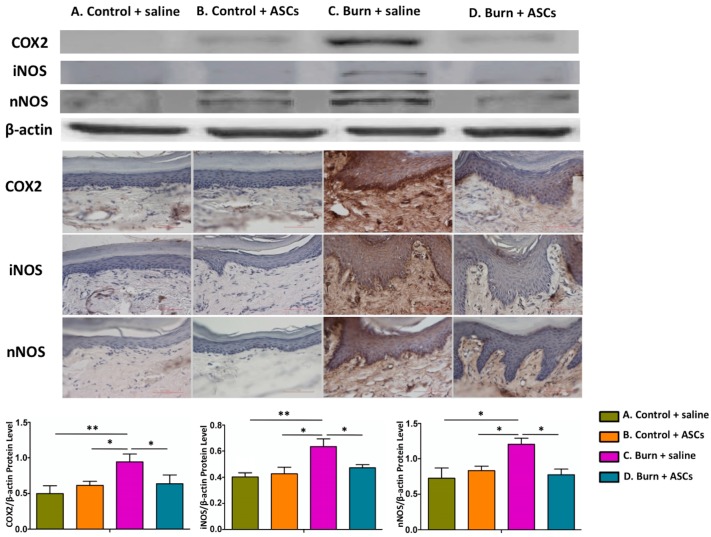
Attenuated local inflammation in post-burn hind paw skin after ASC transplantations. Four weeks after subcutaneous ASC transplantation or saline injection into the post-burn hind paw skin, immunohistochemical staining and western blotting of skin samples revealed significantly increased levels of inflammatory proteins (COX-2, iNOS and nNOS) in Group C, compared with Groups A and D. This indicated that a burn injury could induce local inflammation, which could be attenuated by ASCs. Upper four rows: Representative western blot images. Middle: Representative immunohistochemical staining images. Bottom: Quantitative analyses of the western blotting data. The sample size was *n* > 3 per test and β-actin was used as the internal protein control. (** *p* < 0.01, * *p* < 0.05).

**Figure 5:**
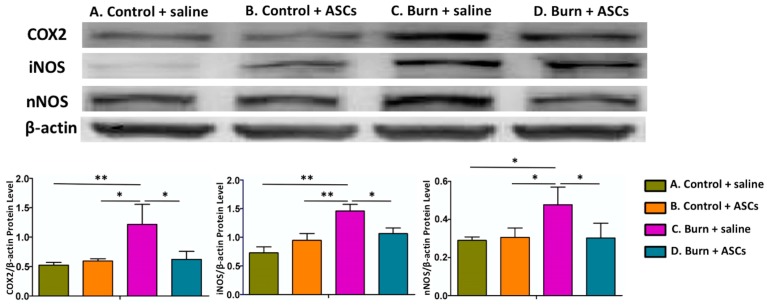
Attenuated central neuroinflammation in spinal cord dorsal horn after ASC transplantations. Four weeks after subcutaneous ASC transplantation or saline injection into burn-injured skin, western blot analyses of dorsal horn specimens showed significantly increased levels of inflammatory proteins (COX-2, iNOS and nNOS) in Group C, compared to Groups A and D, suggesting that a skin burn induces concurrent spinal cord neuroinflammation that is attenuated by ASCs. Upper four rows: Representative western blot images. Lower panel: Quantitative analyses. Sample sizes were >3 per test. β-actin was used as an internal protein control. (** *p* < 0.01, * *p* < 0.05).

At this time, immunohistochemical staining and western blot analyses of hind-paw skin samples demonstrated significantly higher levels of inflammatory proteins (COX-2, iNOS and nNOS) in Group C (Burn + Saline) compared to the other groups (*p* < 0.05) (Figure 4). Western blot analyses of dorsal horn specimens also indicated significantly increased levels of these inflammatory proteins in Group C (*p* < 0.05) (Figure 5). These findings suggest that the burn injury induced local inflammation and spinal cord neuroinflammation, which were attenuated by subcutaneous ASC transplantation.

### 2.2. Ameliorated Inflammation and Apoptosis in the Spinal Cord after ASC Transplantation in Post-Burn Scar

**Figure 6:**
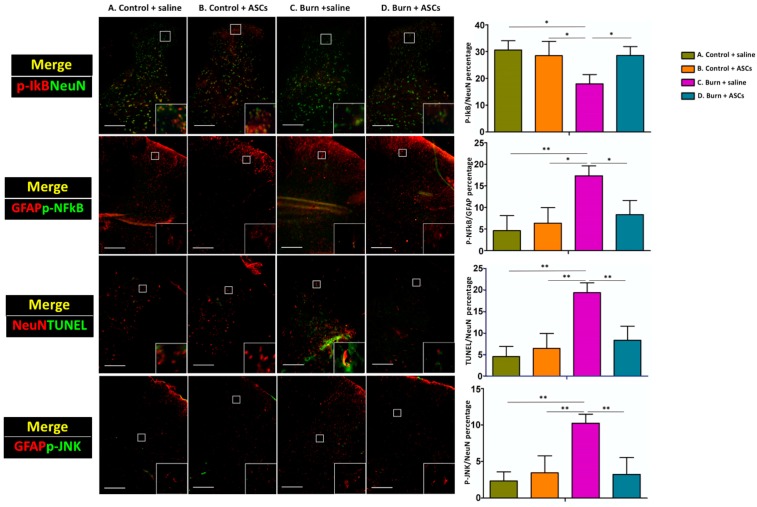
Ameliorated inflammation and apoptosis in the spinal cord after ASC transplantation. Four weeks after subcutaneous ASC transplantation or saline injection into burn-injured skin, quantitative immunofluorescent staining revealed a significant decrease in the total number of NeuN and p-IκB double-positive cells and a significant increase in p-NFκB- and p-JNK-expressing astrocytes in dorsal horn cells from Group C, compared to Groups A and D. Taken together, these results suggest that burn-induced inflammation and apoptosis in dorsal horn cells can be ameliorated by subcutaneous ASC transplantation. (** *p* < 0.01, * *p* < 0.05).

Four weeks after the ASC or saline injection, dorsal horn cells were subjected to double immunofluorescent staining and quantitative analysis to evaluate levels of NeuN (a neuron marker), GFAP (an astrocyte marker—astrocytes are crucial for promoting and maintaining chronic neuropathic pain) [9], p-NFκB (a nuclear transcription factor that regulates inflammation and apoptosis), p-IκB (an inhibitor of NFκB activation), p-JNK (an inflammatory marker) and TUNEL (an apoptosis marker). Notably, the dorsal horn cells from Group C exhibited significantly fewer NeuN/p-IκB double-positive cells (*p* < 0.05), significantly higher TUNEL (*p* < 0.01) and significantly more p-NFκB- and p-JNK-expressing astrocytes (*p* < 0.05) compared with cells from the other groups (Figure 6). These findings indicate that the burn injury to the skin induced inflammation and apoptosis in spinal dorsal horn cells, which was ameliorated by subcutaneous ASC transplantation. 

**Figure 7:**
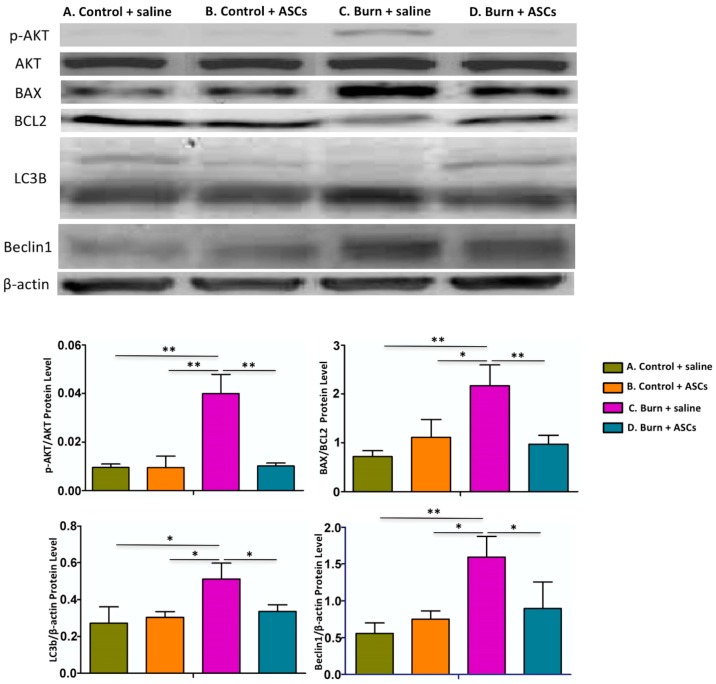
Decreased and apoptosis and autophagy in the spinal cord after ASC transplantation. Four weeks after subcutaneous ASC transplantation or saline injection into burn-injured skin, western blot analyses of dorsal horn specimens revealed significantly increased ratios of p-Akt/Akt and Bax/Bcl-2 and levels of LC3B-II and Beclin 1 in Group C compared to Groups A, B and D. This suggests that the apoptosis and autophagy promoted in the spinal cord were attenuated after ASC transplantation. Data are presented as means ± standard errors of the means. (** *p* < 0.01, * *p* < 0.05).

The dorsal horn cells from Group C also showed significantly elevated p-Akt/Akt and Bax/Bcl-2 ratios compared with those from the other groups in western blot analysis (*p* < 0.05) (Figure 7). Because Akt inhibitors protect neurons from necrosis and apoptosis can be detected as an increased Bax/Bcl-2 ratio [20,21,22], this finding suggests that the burn injury to the skin induced apoptosis in the spinal cell population, which was attenuated by the autologous ASC transplantation.

### 2.3. Decreased Autophagy in the Spinal Cord after ASC Transplantation in Post-Burn Scar

LC3B-II and Beclin 1 levels, which are both related to autophagy [16,17], were significantly elevated in the dorsal horn cells from Group C compared to those from Groups D in western blot analysis (*p* < 0.05), suggesting that the burn injury promoted autophagy in this spinal cell population. Surprisingly, these levels had decreased four weeks after ASC transplantation (Figure 7).

## 3. Discussion

Using a similar model to our previous study with subcutaneous ASC transplantation in post-burn scars, we demonstrated anti-neuroinflammation effects both locally and in the spinal cord that led to the relief of post-burn neuropathic pain and attenuated cell apoptosis.

The differentiation of ASCs and their paracrine effect both contribute to their therapeutic efficacy, which can also be affected by the route and the cell amount administered. Compared to systemic injection and its direct homing effect, local injection results in lower survival of engrafted ASCs, especially in damaged sites [23,24]. Therefore, the paracrine effect is considered to play a major role in treatment by local injection [24].

The exact cell amount that should be administered locally for sufficient engraftment of the ASCs and their subsequent therapeutic effect remains to be clarified. Our previous study found the muscular atrophy attenuation and motor neuron protection results using the same rat model and methods [25]. Therefore, we adopted the same dose in this study—1 × 10^6^ cells.

In other studies, intra-lesion injections of 5 × 10^5^ human ASCs and 1 × 10^6^ autologous ASCs via epineural and intracerebral routes in rats demonstrated the differentiation of ASCs and promotion of neurogenesis at 4 weeks and 14 days after the injections, respectively [26,27]. The local engraftment of autologous ASCs was also found 3 and 8 weeks after intra-lesion injections to tendons and joints of 1 × 10^7^ and 2 × 10^6^ cells in horses and rabbits, respectively [28,29]. The present study used subcutaneous injections of a similar number (1 × 10^6^) of autologous ASCs into the burn scar area, showing engraftment to the dermal layer 4 weeks later.

Chang et al. also found similar thermal thresholds before and after burn injury in their rat model. They thought that was related to the different nociceptors responding to different types of pain—Aβ fibers for light touch and Aδ and C fibers for thermal hyperalgesia [30]. In our previous studies using the same model, we also found the similar results [9,18].

Cyclooxygenase-2 (COX-2) is induced by inflammation and could lead to central sensitization, accompanying chronic pain. NOS synthesizes NO, which is involved in the regeneration of neuropathic pain. Diminished upregulation of nNOS (neuronal NOS) and iNOS (inducible NOS) in the spinal cord and skin were noticed after inhibition of NOS, which resulted in relief of inflammation and neuropathic pain [9]. In this study, increased levels of inflammatory proteins (COX-2, iNOS and nNOS) were found not only in post-burn skin but also in the spinal cord; this was consistent with a previous study that observed concurrent neuroinflammation and subsequent central sensitization [1]. After injecting ASCs into the injury area, we observed significantly decreased post-burn mechanical thresholds and inflammatory proteins in both post-burn skin and dorsal horn cells. This indicated that ASCs injected subcutaneously can ameliorate post-burn pain and central sensitization through anti-neuroinflammation.

Liang et al. showed in macrophages that IL-10 inhibited the production of IL-33 by regulating phosphorylation of the downstream molecule STAT3 and restricting inflammatory responses mediated by IL-33 and the IL-33 receptor ST2 by suppressing the activation of NFκB (a nuclear transcription factor with roles in inflammation and apoptosis [31]) in vitro and in vivo [10]. Activation of the NFκB signaling pathway requires the phosphorylation and degradation of IκB proteins [32]. Notably, the increase we observed in p-IκB expression and the decrease in p-NFκB expression in the dorsal horns in Group D could explain the anti-neuroinflammatory effects of autologous ASCs.

Apoptosis occurs in response to stress or cytotoxicity and can be detected as an increased Bax/Bcl-2 ratio [20,22]; conversely, decreased Bax expression and increased Bcl-2 expression inhibit apoptosis [33]. Furthermore, phosphatidylinositol 3 kinase/Akt activation correlates with cytotoxic cell death and Akt inhibitors have been shown to protect cultured neurons against photodynamically induced necrosis [21]. The decreased p-Akt/Akt and Bax/Bcl-2 ratios in spinal cords in our study may therefore represent the effect of attenuation of apoptosis by autologous ASCs. However, Bax is also a pro-inflammatory cytokine and may be reduced by anti-neuroinflammation processes in rats [34,35]. Thus, the attenuated apoptosis may also be related to the anti-neuroinflammation effect of ASCs.

Several interactions between inflammatory cytokines and autophagy have been identified [36,37], suggesting that the activation of autophagy may be related to the inflammatory responses after the burn injury. The Bcl-2-interacting protein Beclin 1 has been shown to promote autophagy and deficiencies or dysregulation of this protein can lead to neurodegenerative disorders [17,38]. Overexpression of Beclin 1 has been observed after brain injury [39]; this suggests increased autophagy plays a crucial role, exerting neuroprotective effects by removing injured cells and components. LC3B is thought to protect against cell death by promoting autophagy and is considered a marker of autophagy activation when converted from its cleaved form (LC3B-I) to its lipidated form (LC3B-II) because the latter plays a crucial role in the induction of the autophagosome [16].

In this study, we observed promoted autophagy through increased levels of Beclin 1 and LC3B-II in the spinal cord cells after the burn injury. A significant decrease in levels of both markers indicated that the subcutaneous transplantation of ASCs might ameliorate autophagy in the spinal cord.

As we know, our study is the first study adopting ASCs in post-burn pain with an anti-neuroinflammation effect. Besides, we also found the expected neuroprotective aspects of ASCs by showing the attenuations of both apoptosis and autophagy in spinal cord. Since the ASCs were not administered in a systemic way (intravenous or intra-arterial route), the effects registered at sites distant from dermal burns must be due to ASC paracrine effects.

## 4. Materials and Methods

### 4.1. Experimental Design

**Figure 8:**
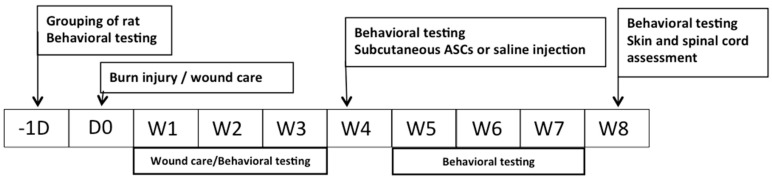
Flowchart of the experimental design. (D, day; W, week).

The animal study protocols were approved by the Institutional Animal Care and Use Committee of Kaohsiung Medical University (IACUC Approval No.: 100048; 3 December 2012). Twenty-four male Sprague–Dawley rats (body weight, 175–200 g; age 6–7 weeks) were randomly obtained from BioLASCO Taiwan Co., Ltd. (Taipei, Taiwan) and housed individually in an animal facility with a 12 h light/dark cycle, a constant temperature of 22 °C and relative humidity of 55%. Standard laboratory rodent chow and sterile tap water were available ad libitum. The rats were randomly divided into four groups (*n* = 6 per group), as follows: Group A received subcutaneous injections of 0.9% saline 4 weeks after sham burns (Control + Saline); Group B underwent subcutaneous transplantations of autologous ASCs 4 weeks after sham burns (Control + ASCs); Group C received subcutaneous injections of 0.9% saline 4 weeks after burn injuries (Burn + Saline); and Group D underwent subcutaneous transplantations of autologous ASCs 4 weeks after burn injuries (Burn + ASCs). Figure 8 illustrates the experimental design.

### 4.2. Burn Injury or Sham Intervention, Wound Care and Behavioral Testing

The rats were anesthetized with a subcutaneous injection of Zoletil 50 (50 μg/g; Virbac Laboratory, Carros, France) prior to creating dermal burn injuries. These were caused by forcing contact (100 g weight) of the right hind paw with a heated metal block for 10 s. The blocks were heated to 25 ± 0.5 °C for rats in Groups A and B (i.e., sham burn injuries) and to 75 ± 0.5 °C for rats in Groups C and D (i.e., third-degree scald burn injuries, which were treated daily with silver sulfadiazine cream for approximately 3 weeks until the wounds healed).

Mechanical and thermal thresholds were measured with the paw withdrawal threshold and paw withdrawal latency tests, respectively, as described in our previous reports [9,18]. For the paw withdrawal threshold test, the hind paw was placed on a metal mesh and a mechanical stimulus (a 2-mm diameter metal rod) was applied to stimulate the plantar surface with a pressure increasing at a rate of 2.5 g/s until the rat withdrew the paw; the exact pressure at which this occurred was recorded (in grams) using a Dynamic Plantar Aesthesiometer (Ugo Basile, Varese, Italy). For the paw withdrawal latency test, an infrared radiant heat source was positioned beneath the plantar surface of the hind paw and the time until the rat withdrew the paw was measured (in seconds). Each measurement was repeated six times at 10-min intervals. All tests were performed 1 day before and 1 week after the burn injuries and at 1-week intervals thereafter for an additional 8 weeks.

### 4.3. Isolation, Surface Marker Analysis, Labeling and Transplantation of the ASCs

The isolation, labeling and transplantation procedures for the ASCs were similar to those in our previous study [25]. Autologous adipose tissues were harvested from the left inguinal areas of the rats and cut into pieces (approximately 1 × 1 × 2 cm in size) using scissors. These were washed in phosphate-buffered saline (PBS) and digested with 0.075% collagenase (37.5 mg/mL; Sigma-Aldrich, St. Louis, MO, USA) in PBS at 37 °C with constant agitation for 30 min. The digested samples were then centrifuged (800× *g*, 10 min) to separate the supernatant containing the mature adipocytes from the pellets. The pellets were suspended and filtered through a 40-mm cell strainer (BD Biosciences, Franklin Lakes, NJ, USA) and then washed and incubated in Dulbecco’s modified Eagle’s medium (GIBCO/Invitrogen Corporation; Carlsbad, CA, USA) supplemented with 2 mmol/L *N*-acetyl-l-cysteine (Sigma-Aldrich) and 0.2 mmol/L l-ascorbic acid 2-phosphate (Sigma-Aldrich) at 37 °C. The medium was changed every 3 days.

**Figure 9:**
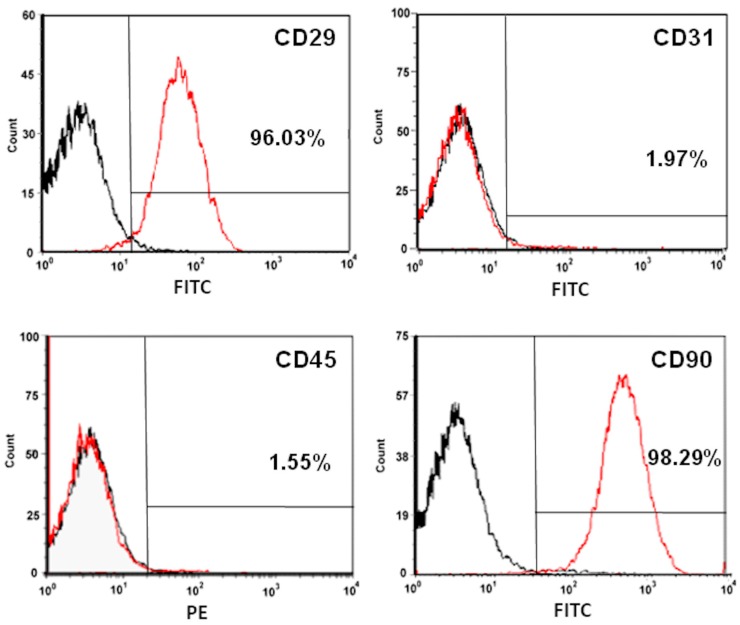
ASC surface marker expression detected by flow cytometric analysis. Surface marker analysis of cells from cultured rat adipose tissues using flow cytometry. ASCs were defined as CD29^+^/CD31^−^/CD45^−^/CD90^+^. The percentages are the mean values from three experiments. The red line represents positive staining cells and the black line stands for iso-type-matched antibody control.

After 1 week in culture, the cells were trypsinized, washed twice with PBS, blocked for 1 h at room temperature and incubated overnight at 4 °C with a solution of fluorescein isothiocyanate (FITC)-conjugated or R-phycoerythrin (PE)-conjugated antibodies in PBS. A flow cytometry system (LSR II, BD Biosciences) was used to examine the expression of surface markers on subcultured cells collected during the third passage. The following antibodies were used: FITC anti-mouse/rat CD29 (102206, BioLegend, San Diego, CA, USA), FITC anti-rat antibodies CD90 (206106, BioLegend), FITC mouse anti-rat CD31 (MCA1334, Serotec, Raleigh, NC, USA) and PE anti-rat CD45 (202207, BioLegend). The first two of these antibodies were used as positive antigen markers, the second two as negative antigen markers. FITC rat immunoglobulin (Ig) G2b, k isotype control and PE rat IgG2a, k isotype control (400508, BioLegend) were used as staining controls and to ensure accurate measurements. ASCs were defined as cells that were CD29^+^/CD31^−^/CD45^−^/CD90^+^ on flow cytometric analyses (Figure 9).

ASCs were labeled with chloromethylbenzamido (C7000, CellTracker CM-DiI; Invitrogen/Life Technologies, Carlsbad, CA, USA) according to the manufacturer’s instructions. Labeled fourth-passage cells were visualized using fluorescence microscopy prior to harvesting for treatment (Figure 2a). Four weeks after the injury or sham-injury intervention, rats in Groups B and D each received a subcutaneous injection of a 0.4 mL aliquot (1 × 10^6^ cells) of ASCs into the scar area of the right hind paw, administered using a BD Ultra-fine II 1 mL insulin syringe with a 27-gauge needle. The engraftment of cultured ASCs into the post-burn dermis was identified by the co-localization of CM-Dil (red)/CD90 (green) staining and DAPI (4,6-diamidino-2-phenylindole) nuclear counterstaining (blue) via fluorescence microscopy, 4 weeks after the transplantation of cultured ASCs (Figure 2d,e).

On the other hand, each rat in Group A and C was injected with 0.4 mL 0.9% normal saline subcutaneously four weeks after the injury or sham-injury intervention.

### 4.4. Immunohistochemical Staining for Cyclooxygenase-2, Inducible Nitric Oxide Synthase and Neuronal Nitric Oxide Synthase

Four weeks after the ASC or saline injection, the rats were sacrificed using an overdose of Zoletil 50. Skin from the right hind paw was excised, fixed in formalin, embedded in paraffin and 4-μm-thick specimens were mounted on saline-coated slides, deparaffinized and rehydrated in a series of graded alcohol solutions. This was followed by antigen retrieval in citrate buffer (pH 6.0; 0.1 mol/L), heating to 121 °C for 10 min and cooling to room temperature. To quench the endogenous peroxidase activity, the sections were further incubated for 5 min in 3% H_2_O_2_. Non-specific binding was blocked by incubation with 5% goat serum in PBS for 30 min. The blocked sections were incubated overnight at 4 °C with rabbit polyclonal antibodies against cyclooxygenase (COX)-2 (#12282, 1:200 dilution; Cell Signaling, Danvers, MA, USA), iNOS (ab15323, 1:200; Abcam, Cambridge, MA, USA) and nNOS (ab76067, 1:200; Abcam), rinsed and incubated with horseradish peroxidase-conjugated secondary antibodies for 30 min at room temperature. The slides were exposed to the colorimetric reagent 3,3-diaminobenzidine for 5 min, counterstained for 1 min with Mayer’s hematoxylin and mounted for evaluation.

### 4.5. Double Immunofluorescence Labeling and TUNEL Assay

We excised the L3–L5 segments of the lumbar spinal cords of all rats at 4 weeks after cultured ASC transplantation or subcutaneous saline injection. Dorsal horn tissues were isolated and prepared as described in a preceding article [9]. Double immunofluorescence detection was performed by incubating tissues overnight at 4 °C with one of the following antibody combinations: polyclonal phospho-inhibitor of κB (p-IκB; 1:100 dilution; Cell Signaling) and monoclonal NeuN (MAB377, a neuronal cell marker; 1:1000; Millipore, Temecula, CA, USA); monoclonal glial fibrillary acidic protein (610565, GFAP, an astrocyte marker; 1:1000; BD Biosciences) and polyclonal phospho-nuclear factor κB (#3033, p-NFκB; 1:100, Cell Signaling); and monoclonal GFAP and phospho-Jun N-terminal kinase (#4668, p-JNK; 1:100; Cell Signaling). The tissues were subsequently stained with the secondary antibodies Cy3-conjugated goat anti-rabbit (AP187C, red; Millipore) and Alexa Fluor 488-conjugated goat anti-mouse (A11001, green; Invitrogen).

Apoptotic cell death was detected by a TUNEL (terminal deoxynucleotidyl transferase-mediated dUTP nick end labeling) assay using an ApopTag Fluorescein in Situ Apoptosis Detection Kit S7110 (Millipore), according to the manufacturer’s instructions. The treated sections were incubated with a NeuN primary antibody (MAB377, 1:1000; Merck Millipore, Bedford, MA, USA) at 4 °C and then with a Cy3-conjugated anti-mouse IgG secondary antibody (Merck Millipore) at room temperature for 1 h, rinsed three times with PBS for 5 min and mounted with DAPI-containing medium. A fluorescence microscope (DMI6000; Leica Microsystems, Wetzlar, Germany) was used to obtain images and quantitative results.

### 4.6. Western Blot Analyses

Separated skin and L3–L5 dorsal horn specimens from all rats were processed using a western blotting protocol described previously [9], using antibodies against the following proteins: COX-2 (#12282, 1:1000 dilution; Cell Signaling), iNOS (ab15323, 1:1000; Abcam), nNOS (ab76067, 1:1000; Abcam), Akt/protein kinase B (#4685, 1:1000; Cell Signaling), p-Akt (#4060, 1:1000; Cell Signaling), B-cell lymphoma 2 (ab59348, Bcl-2; 1:1000; Abcam), Bcl-2-associated X protein (50599-2-Ig, Bax; 1:1000; Proteintech Group, Chicago, IL, USA), β-actin (A5441, 1:20000; Sigma-Aldrich), LC3B (1:1000; Cell Signaling) and Beclin 1 (#2775, 1:1000; Cell Signaling). The membranes were visualized by Bio-Rad ChemiDoc MP and band intensity was quantitated by Quantity One 1-D Analysis software (Bio-Rad Laboratories Inc., Hercules, CA, USA).

### 4.7. Statistical Analysis

SPSS software (version 14.0; SPSS, Inc., Chicago, IL, USA) was used for the statistical analysis. The mean values and standard deviations of numerical data were calculated as shown in the figures and figure legends. Western blot measurements were tested using one-way analysis of variance and Tukey pairwise comparison. A *p*-value <0.05 was considered statistically significant.

## 5. Conclusions

In a rat model, autologous ASC subcutaneous transplantation in post-burn scars elicited anti-neuroinflammation effects locally and in the spinal cord that might be related to the relief of post-burn neuropathic pain and attenuated cell apoptosis. Autophagy promoted in the spinal cord after burn injury was also ameliorated. Thus, ASC transplantation for post-burn scars shows the potential for promising clinical benefits.
